# Gut microbiota and its implications for psychiatry

**DOI:** 10.1192/j.eurpsy.2021.1258

**Published:** 2021-08-13

**Authors:** A. Matas Ochoa, A. Rodriguez Quiroga, R. Martinez De Velasco, P. Nava Garcia, C. Banzo Arguis, I. Moreno Alonso

**Affiliations:** Psychiatry, Hospital Universitario Infanta Leonor, Madrid, Spain

**Keywords:** microbiota, gut, brain, psychiatry

## Abstract

**Introduction:**

In recent years there has been increasing interest in knowing the function of the microbiota, especially its role in the gut-brain axis. The microbiota is the set of millions of microorganisms that coexist in a symbiotic way in our body and are located in the digestive tract mainly. Numerous evidences show that the microbiota could modulate the information directed to the brain and therefore the pathogenic basis of numerous psychiatric and neurological disorders.

**Objectives:**

A better understanding of the microbiota and its interaction with the brain and mental health.

**Methods:**

Review of recent literature about the implications of the gut microbiota in psychiatry.

**Results:**

The connection between the microbiota and the central nervous system (gut-brain axis) occurs through the vagus nerve, the systemic pathway (through the release of hormones, metabolites and neurotransmitters) and the immune system (through the action of cytokines). Changes in the microbiota are associated not only with gastrointestinal diseases, but also with disorders such as depression, anxiety, autism, anorexia, attention deficit and hyperactivity, Alzheimer’s disease and Parkinson’s disease. As some research indicates, changes in diet and composition of the microbiota can reduce the risk of suffering these diseases or reduce their symptoms. Other therapeutic alternatives postulated are the use of probiotics or fecal microbiota transplantation.

**Conclusions:**

Despite growing interest in the microbiota in the last few years, little is known about the mechanisms underlying this communication. More research is expected to contribute to the design of strategies that modulate the gut microbiota and its functions in order to improve mental health.

